# Coupled methods for detecting fatigue damage in thin-walled composite structures with different types of notches

**DOI:** 10.1038/s41598-026-48942-0

**Published:** 2026-04-14

**Authors:** Patryk Różyło, Jakub Paśnik, Marek Barski, Adam Stawiarski, Paweł J. Romanowicz, Małgorzata Chwał

**Affiliations:** 1 Department of Machine Design and Mechatronics, Faculty of Mechanical Engineering, Lublin University of Technology, Nadbystrzycka 36, 20-618 Lublin, Poland; 2 Department of Machine Design and Composite Structures, Faculty of Mechanical Engineering, Cracow University of Technology, Warszawska 24, 31-155 Cracow, Poland

**Keywords:** Fatigue, NDT methods, Damage, Notches, CFRP composites, Experimental study, Engineering, Materials science

## Abstract

The paper focused on the fatigue testing of thin-walled composite structures with notches. The fatigue experiments were supported by non-destructive diagnostic techniques, including thermography, acoustic emission, and digital microscopy. The study analysed the influence of notch geometry on the initiation and evolution of damage in CFRP (carbon fiber reinforced polymer) specimens with a [0/45/-45/90]_s_ lay-up. All specimens were tested under identical fatigue loading conditions. Within the scope of the research, quantitative characteristics of thin-walled structures under fatigue loading were developed, and the recorded damage mechanisms were examined qualitatively. The combination of interdisciplinary research methods made it possible to carry out a thorough analysis of composite structure behaviour. Tests involving the complete failure of the specimens were not conducted. Instead, the study focused on the detection and monitoring of early stages of damage within the low-cycle fatigue regime. The application of coupled diagnostic methods for detecting and monitoring fatigue damage made it possible to investigate the differences in damage initiation and development between specimens with U-shaped and V-shaped notches.

## Introduction

The fatigue phenomenon involves the progressive damage of a material’s structure under cyclic loading, leading to cracks and ultimately catastrophic loss of load-carrying capacity^1–4^. The classical fatigue testing on metals, initiated in the 19th century by Wӧhler, laid the foundations for the research methodology, including the concept of S–N curves and the concept of fatigue limit^5^. The fatigue strength of materials is one of the key criteria for their use in mechanical engineering, aerospace, and energy. Almost every branch of industry relies on fatigue testing of the materials they utilize.

Fatigue damage caused by cyclic fatigue loads occurs as a result of formation of microcracks due to local stress concentrations and their subsequent gradual propagation in the material. The main causes of this phenomenon include the presence of structural discontinuities, such as surface defects, inclusions, porosity, phase boundaries or sharp shape changes in the object, which contribute to local stress growth^1,6,7^. Anisotropy of properties also plays an important role in composite materials, leading to uneven load distribution between fibers and matrix^8^. Furthermore, environmental conditions such as humidity, temperature, chemical or atmospheric factors can accelerate the degradation process and reduce fatigue performance^9^.

A number of characteristic damage mechanisms are reported for fiber composites, which develop under fatigue loading. The most common ones include^10^: delamination – a connection failure between individual layers of laminate, which reduces the stiffness of the structure and facilitates the propagation of subsequent damage^11^; matrix cracking—initiation and development of microcracks in the resin, promoting uneven load transfer to the fibers, fiber breakage—local or extensive fiber breaks, leading to a significant loss of load-carrying capacity along the direction of their arrangement; fiber–matrix debonding—adhesion loss between fibers and the matrix, causing a reduction in effective stress transmission.

As these mechanisms often coexist and interact, the fatigue process in composites is becoming more complex. The analysis and identification of these mechanisms are an essential part of research, enabling both the assessment of in-service durability and the development of predictive models.

^9,12^. As materials science and mechanical engineering have developed, particular attention has been paid to composites, which are characterized by high levels of specific strength and stiffness compared to conventional materials. Composite materials, especially fiber reinforced composites, have been widely utilized in aerospace and automotive applications^13^. However, their fatigue failure mechanisms are much more complex than in the case of metals, which results from the anisotropy of mechanical properties, the presence of many types of degradation, and their dependence on environmental factors^8^. The literature also shows that the presence of notches and technological holes can significantly accelerate the fatigue degradation process, which is demonstrated by experimental results acquired for multilayer laminates^6^ and composite panels with holes^14^. It is important to properly prepare specimens and to control test conditions, as local defects can significantly affect measurement outcomes^15^.

In recent years, there has been rapid growth in the use of non-destructive diagnostic techniques such as thermography, acoustic emission, digital image correlation, and computed tomography, which can monitor the development and evolution of damage during fatigue cycles^14,16^.

The first two mentioned techniques above have been used in this work to assess the type and nature of fatigue damage. Infrared thermography demonstrates a great potential in detecting damage in layered laminates because of its high inspection speed, the real-time measurement capability, e.g., during fatigue testing, and the visualization of the complete damage profile. It is a simple technique to use and does not require large expenditures during testing, although the equipment needed for this is quite expensive. The effectiveness of this method reduces with the increase in the thickness of the tested material and is most effective for thin samples, which is why it is well suited for analyzing the behavior of laminates. In practice, one of two modes is used: passive mode (monitoring natural temperature changes under load) and active mode (thermal stimulation, e.g., through light or heat pulses). Currently, infrared thermography is used to detect voids, delamination, debonding, fiber and matrix cracking, and impact damage^17–19^. Acoustic emission (AE) is another non-destructive material testing technique that enables observation of the timing of damage initiation and propagation. The AE technique exploits the fact that damage development processes in a material (for composites, matrix or fiber cracking, delamination, fiber pull-out, debonding) generate high-frequency elastic waves, starting at 100 kHz and reaching 1 MHz for the latest AE equipment^20^. Signals recorded by piezoelectric sensors not only enable damage detection, but also allow for its localization and classification according to the mechanism^16,21–24^.

Research results show that despite the complexity of fatigue degradation processes, it is possible to model and predict the fatigue life of composite structures based on advanced numerical and experimental methods^25–27^. Conducting innovative fatigue research in this area, including both the development of new fiber configurations and the improvement of diagnostic methods, is the foundation for the further development of materials engineering. This is a high-dynamic area of great application significance, supporting the need to intensify research on fiber composites in the context of their behavior under fatigue loads. Recent advances in this area show that modern non-destructive techniques have significantly improved the ability to detect early‑stage fatigue degradation in fibre‑reinforced composites^26^. State‑of‑the‑art research demonstrates that high‑resolution passive thermography enables real‑time monitoring of thermal signatures associated with matrix cracking, delamination and fibre–matrix debonding, while advanced acoustic emission systems provide more accurate identification and localisation of damage mechanisms through energy‑based signal analysis. Contemporary studies emphasize the combined use of these methods as a consistent approach to characterising the initiation and evolution of fatigue damage in composites.

The presented study is mainly focused on two issues related to material fatigue. The first concerns the determination of the fatigue damage behaviour of the investigated multilayer composite specimens weakened by notches of varying intensity. For this purpose, two different notches—moderate U-shaped notch and sharp V-shaped notch – are selected. Such notches are commonly used in the literature for fatigue testing of different materials^28,29^. It is shown that, depending on the notch tip radius, the stress concentration factor for such notches can vary significantly—even by several 100%. The second key issue examined in the study is the use of non-destructive methods for detecting and monitoring fatigue damage during fatigue experiments. In particular, the study focuses on the possibilities of detecting and analysing damage in specimens in which the notch intensity differed significantly from each other. For this reason, fatigue tests are performed at a relatively high tensile load (relative to the static tensile test) and are carried out to a fixed number of cycles.

The application of non-destructive methods during fatigue testing is of great importance because the results obtained using only a testing machine do not permit a direct, comprehensive evaluation of the specimens’ behaviour, particularly in the context of fatigue failure behaviour. Testing techniques utilising acoustic emission signals enable the precise determination of the moment of damage initiation, while passive thermography allows the identification of damage localisation by analysing temperature changes. Consequently, both techniques directly contribute to assessing the behaviour of specimens with different notches. These non-destructive testing techniques enable the evaluation of parameters that differentiate the behaviour of specimens featuring various notch types.

In this study, composite flat specimens with notches are tested. The effect of notch shape and size on the fatigue behaviour of thin-walled composite specimens is investigated. In addition, non-destructive techniques are used to assess the degree of structural damage during testing, i.e., passive infrared thermography and acoustic emission. Furthermore, active thermography inspection is performed prior to testing to assess the quality of specimen fabrication. After the tests, the composite specimens are examined using an optical microscope to assess the observed failure forms. Tests involving the complete failure of the specimens are not conducted during the research – only partial damage progression observed during cyclic loading is evaluated.

The conducted research demonstrates that the use of above-described multidisciplinary research methods allows for the quantitative and qualitative determination of damage in multilayer composite structures weakened by notches of varying degrees of notch intensity. This issue is particularly important in structural health monitoring of structures requiring high safety levels (e.g., aviation). Appropriate calibration of the presented methods also allows for real-time damage assessment.

## Materials and specimen preparation

The subject of study was thin-walled composite specimens with two different notches. In order to assess the possibility of damage detection analysis for different stress intensity levels, V- and U-type notches were selected. Such notches produce distinct fatigue behaviours. A V-shaped notch, which belongs to the group of sharp notches, causes a severe stress concentration at the tip of the notch what generally reduces the fatigue life. A U-shaped notch, which have larger root radius, has better fatigue performance due to lower stress concentration around the notch.

**Fig. 1:**
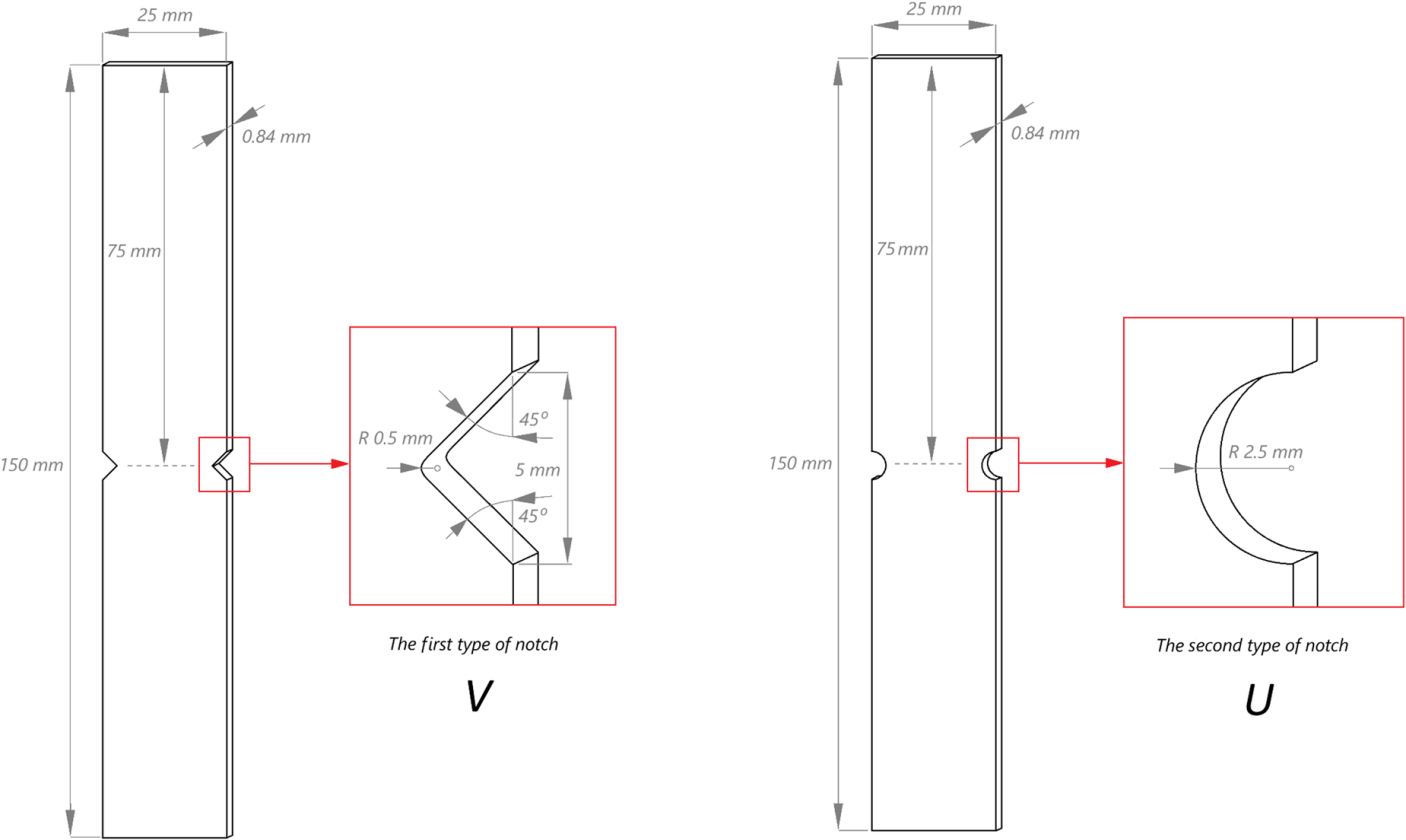
Dimensions of test specimens.

The test specimens have a constant nominal cross-section. The dimensions of the analysed specimens are demonstrated in Fig. 1.

**Fig. 2:**
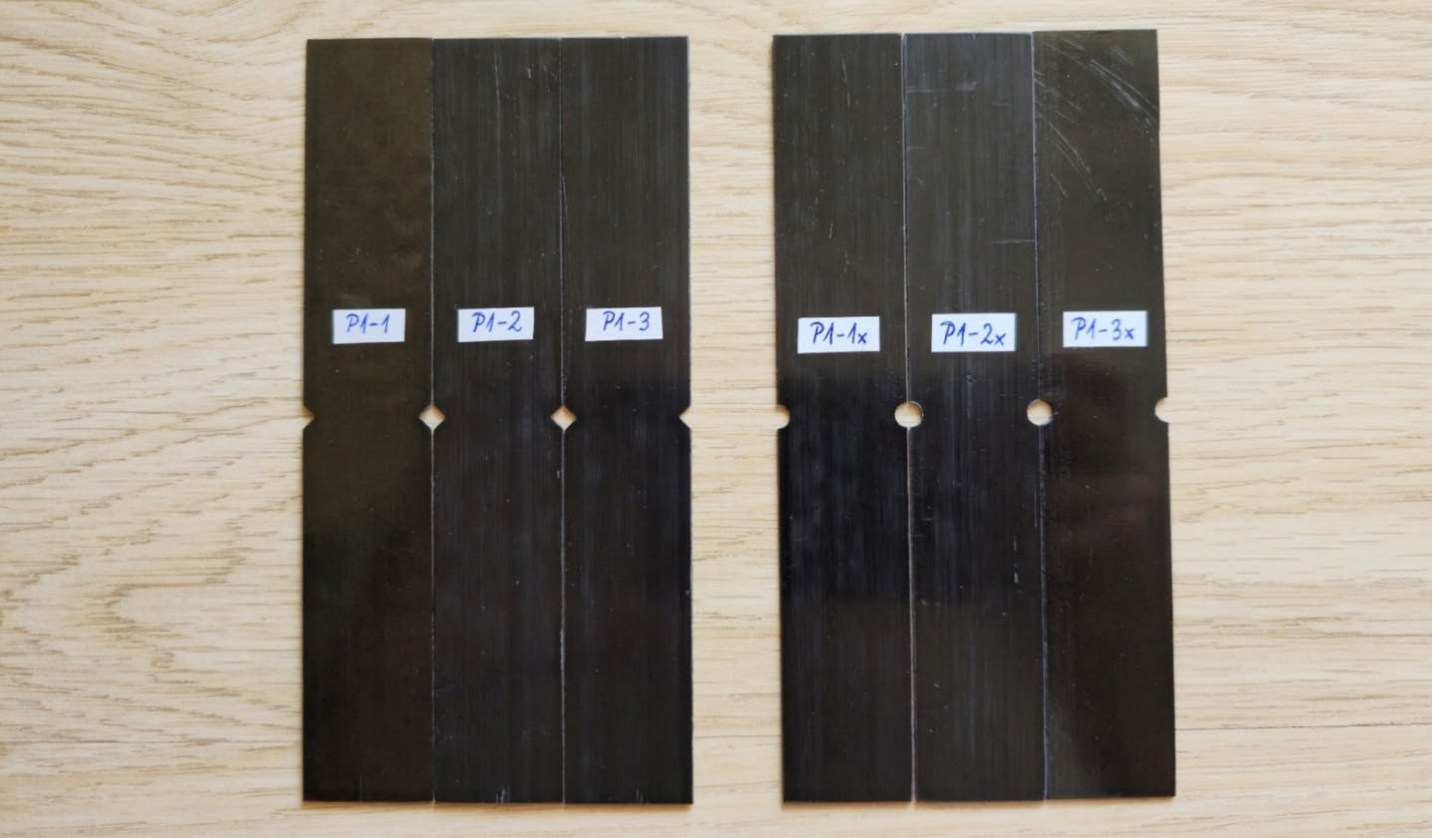
Test specimens for experimental testing.

Three test specimens were prepared for each notch type. The specimens used for the experiment are shown in Fig. 2. Specimens with V-shaped notch were marked as P1-1, P1-2 and P1-3, while specimens with U-shaped notch were marked as P1-1x, P1-2x and P1-3x.

The specimens were manufactured using the autoclave technique and cut using waterjet technology. The diameter of the cutting jet was 1 mm, which ensured the highest possible precision with the cutting tolerance equal to ± 0.1 mm. They were cut from composite structure which have been presented in a variety of scientific publications^30–32^. The geometric parameters of the specimens undergoing experimental testing were selected to ensure the tests were performed correctly, particularly with regard to notch geometry. All the test specimens had the same lay-up.

**Fig. 3:**
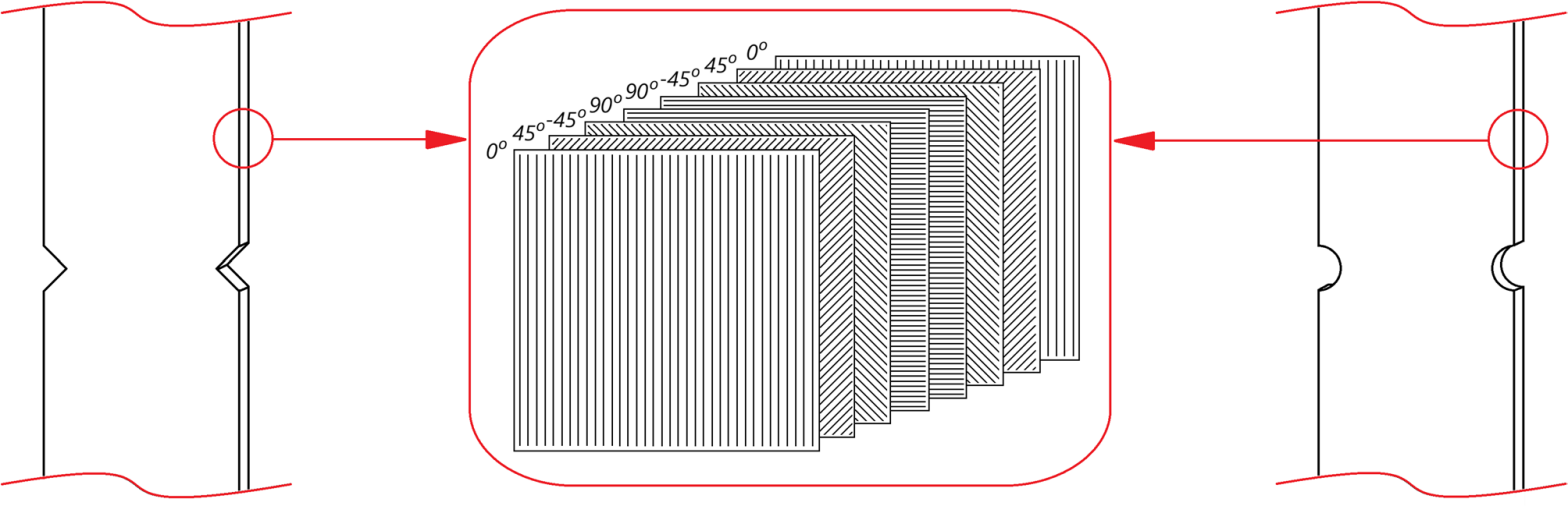
Stacking sequence of composite specimens.

[0°/45°/− 45°/90°]_s_, as shown in Fig. 3, with symmetrical configuration in stacking sequence.

The material properties, determined on the basis of static material tests, are presented in publications^31,32^. The properties were determined in accordance with the relevant standards: tensile test (ISO 527), compression test (ISO 14126) as well as shear test (ISO 14129) – references to standards are included in^33^. Comprehensive details on how to determine the properties of CFRP composites are presented in a publication that describes the methodology in detail^33^. Table 1 presents the material properties in terms of mechanical and strength parameters.

**Table 1:** Material properties of CFRP composite.

Tensile strength F_TU_ [MPa]		Young’s modulus E [MPa]		Poisson’s ratio ν_12_	Shear strength F_SU_ [MPa]	Kirchhoff’s modulus G_12_ [MPa]	Compression strength F_CU_ [MPa]	
0°	90°	E_1_ (0°)	E_2_ (90°)	–	± 45°	–	0°	90°
2220.7	49	143528.5	5826.3	0.36	83.5	3845.5	641	114

### Research methodology

**Fig. 4:**
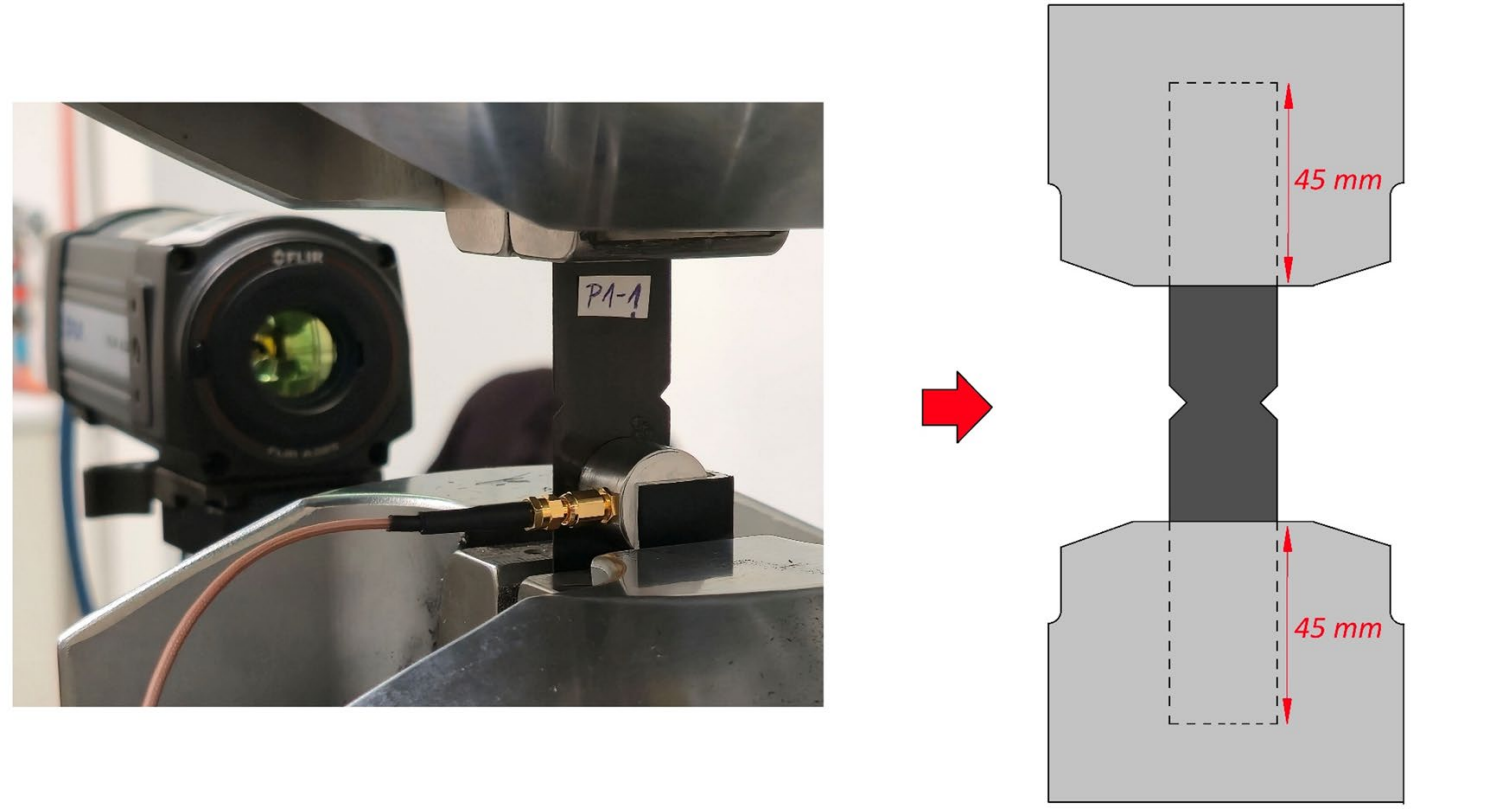
Fatigue testing machine.

The research focused on tests that make it possible to analyse the development of fatigue damage, enabling the distinction of damage associated with different notch geometries. During the study, fatigue tests were carried out using additional non-destructive testing equipment to record defects appearing in the specimens. These tests were performed on CFRP composite specimens featuring both types of notch, with the aim of assessing the impact of notch shape on fatigue behaviour and the defects observed during cycling loading. The fatigue tests were conducted using an MTS Landmark 370 servo-hydraulic testing machine (maximum load capacity 100 kN) for static and fatigue testing^34^. During the tests, dedicated MTS fatigue machine grips (model 647.10 A) were used, applying a clamping force of 2000 psi (13.79 MPa). The load applied during the tests was recorded using an MTS force transducer controller (model 661.20 H-03). The experimental data (e.g., applied force, number of cycles, and displacement) were recorded using MTS Flex Test™ 40 Station Manager software. Figure 4 shows the machine used for static and fatigue testing.

During the tests, it was ensured that the specimens were correctly mounted in the grips of the fatigue testing machine. In order to prevent failure of specimens in the grips or their surroundings, the specimens were mounted in machine grips at a length of 45 mm that covers more than 75% of the available jaw face length. Generally, it is recommended to use tabs on the both ends of the specimens, mainly in the case of composite or brittle materials. However, during preliminary fatigue tests of the described above notched specimens, fatigue damage was observed only in the area of the notches and there was no observable premature failure at the grip faces. Based on this observation as well as the approaches used in the literature^28,29^, the experimental fatigue tests were carried out for specimens without additional tabs at the ends.

**Fig. 5:**
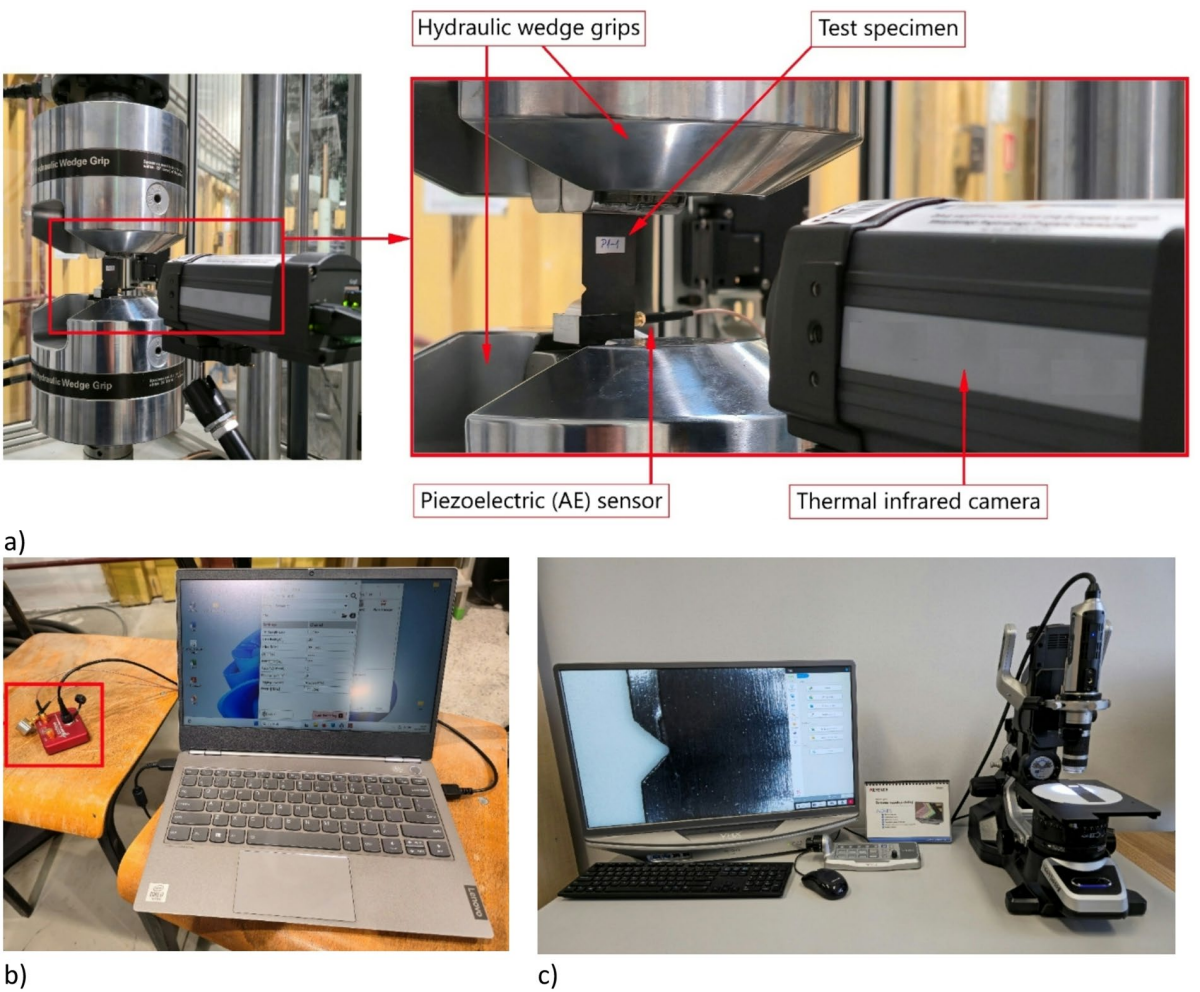
Non-destructive testing equipment: (**a**) a hydraulic wedge grip with a test specimen and a thermographic camera, (**b**) a single-channel system for testing acoustic emissions with PC, (**c**) equipment setup for optical inspection of damage using a digital microscope.

Additional testing equipment was used to record damage initiation and evolution during fatigue testing. For this purpose, thermography (mainly passive) and acoustic emission methods were employed. For passive thermography, which involves monitoring temperature distribution on the surface of the tested object without external thermal stimulation, a FLIR A325 thermal infrared camera (FLIR Systems, Wilsonville, USA) with IrNDT v1.7.2 and ThermaCAM Researcher Pro 2.10 software were used^18,35^. The camera’s specifications were as follows: frame rate of 60 Hz; spectral range of 7.5–13 μm; thermal sensitivity < 50 mK; and temperature range of − 20 to + 350 °C. The thermal infrared camera had a 320 × 240 pixel detector, enabling higher accuracy and the ability to show more detail at longer distances. Tests using passive thermography enabled information about the temperature parameter to be acquired in real time, as well as visualisation of selected thermal changes observed on the test specimens. During passive thermography, temperature measurements were acquired at a frequency of 1 Hz throughout the fatigue test. In this approach, the temperature increase ΔT is defined as ΔT = T_max_ − T_ref_, where T_ref_ represents the initial average surface temperature of the specimen recorded at the beginning of the fatigue test, and T_max_ corresponds to the maximum temperature detected across the specimen surface. Active thermography was also used to obtain information about the condition of the specimens in terms of thermal changes before and after fatigue testing. The specimens were heated with a 400 W halogen lamp for 10 s, with a total analysis and data collection time of 40 s at a frequency of 30 Hz. The thermal response of the specimens was recorded during the heating and cooling processes. The SpotWave 201 system was used for acoustic emission testing^36^. This fully functional AE measurement system is compliant with EN 13477-1. This portable, single-channel device allows acoustic emission data to be recorded and processed (AE event processing), as well as transient waveforms. The system’s input range is ± 50 mV (100 dBAE) and its maximum sampling rate is 2 MHz (16 bits), with the option to reduce the frequency. The tests were conducted at a sampling rate of 0.5 MHz. The acoustic emission data were recorded using the spotWave acquisition and VisualAE software. The system was also equipped with a VS150-L piezoelectric sensor (passive piezoelectric AE sensor). This sensor is ideal for inspecting non-ferromagnetic objects, such as composites and aluminium alloys (frequency range f_Peak_ [kHz]: 100 to 450, operating temperature [°C]: − 50 to + 100). Additional equipment is shown in Fig. 5.

The research equipment shown in Figs. 4 and 5 formed the basis of the research activities. In addition to the aforementioned equipment used for experimental testing, a digital microscope was also employed. Using digital microscopy enabled comprehensive observation of the defects (damage) that appeared during the fatigue tests. For this purpose, a Keyence VHX-970 F digital microscope (Fig. 5c) with a mobile recording head was used [36]. A VH-Z20T lens with a maximum magnification of 200x was used for observation. In addition, damage forms were recorded using a VHX-7020 camera module, a VHX-A97FP console and a VHX-S600E multi-angle observation system. As part of the digital microscopy analysis, the damage forms of the test specimens were recorded using the depth of field function to enable more thorough observation of the defects resulting from the fatigue tests. Additionally, 3D visualisation of the damaged areas of the test specimens was performed. Digital microscopy made it possible to assess the nature of fatigue damage.

An additional static test was initially performed to determine the maximum load value (12.5 kN), in order to correctly specify the fatigue parameters. Following a series of preliminary fatigue tests, the parameters at which fatigue tests could be effectively performed were determined. The parameters that were established following preliminary tests were as follows: f = 10 Hz (frequency); stress ratio *R* = 0.1, F_max_ = 10 000 N (maximum tensile load); The maximum fatigue load was established in preliminary tests to ensure stable, repeatable cycling that produced clear early‑damage signatures detectable by passive infrared thermography and acoustic emission. The experiment was designed to monitor damage initiation and evolution rather than to determine fatigue life; therefore, the number of cycles was limited to 10 000 cycles, providing a controlled degradation range with sufficient observability of thermographic and AE responses. Each fatigue test was preceded by tensioning the specimens to mean load F_m_ with a loading rate of 10 kN/min. As part of the fatigue testing, sinusoidal force excitation was applied. According to the established parameters, a series of research tests could be carried out to analyse the behaviour of specimens of types P1-1, P1-2 and P1-3 – V-shaped notch, as well as specimens P1-1x, P1-2x and P1-3x – U-shaped notch (Fig. 1).

## Research results

As part of the research tests, it was important to identify any damage to thin-walled composite structures during fatigue testing. Two types of notch were analysed in test specimens designated.

P1-1, P1-2 and P1-3 (V-shaped notch) and P1-1x, P1-2x and P1-3x (U-shaped notch).

Initially, a brief analysis of the degradation of specimen stiffness was conducted. The parameter analysed was the specimen’s equivalent stiffness, which is defined as the quotient between the doubled amplitude of the applied load (F_max_-F_min_) and the specimen’s elongation (u_max_-u_min_). The maximum elongation of the specimen u_max_ was determined at the same time point at which the maximum load F_max_ occurred. Similarly, the minimum elongation of the specimen u_min_ was determined at the point at which the specimen was tensioned by the minimum load F_min_. In order to limit the number of points and minimise errors resulting from the sensors’ sampling frequency, the equivalent stiffness E was determined for a specific range of cycles within the n–m interval. It was assumed that this interval was 75 cycles. The equivalent stiffness of the specimen was calculated using the following formula (1):

1 $$\:E\left(n-m\right)=\frac{F_{max}\left(n-m\right)-F_{min}(n-m)}{u_{max}\left(n-m\right)-u_{min}(n-m)}$$

The normalised stiffness E_n_ for the cycle range n-m was calculated as follows (2):

2 $$\:{E}_{n}\left(n-m\right)=\frac{E\left(n-m\right)}{{E}_{0}\left({n}_{0}\right)}$$

Where: E_0_(n_0_) is the stiffness assumed as the initial stiffness and calculated for cycle n_0_. For the calculations, it was assumed that n_0_ equalled 500 cycles, i.e. the number of cycles after which the load on the specimen stabilised.

**Fig. 6:**
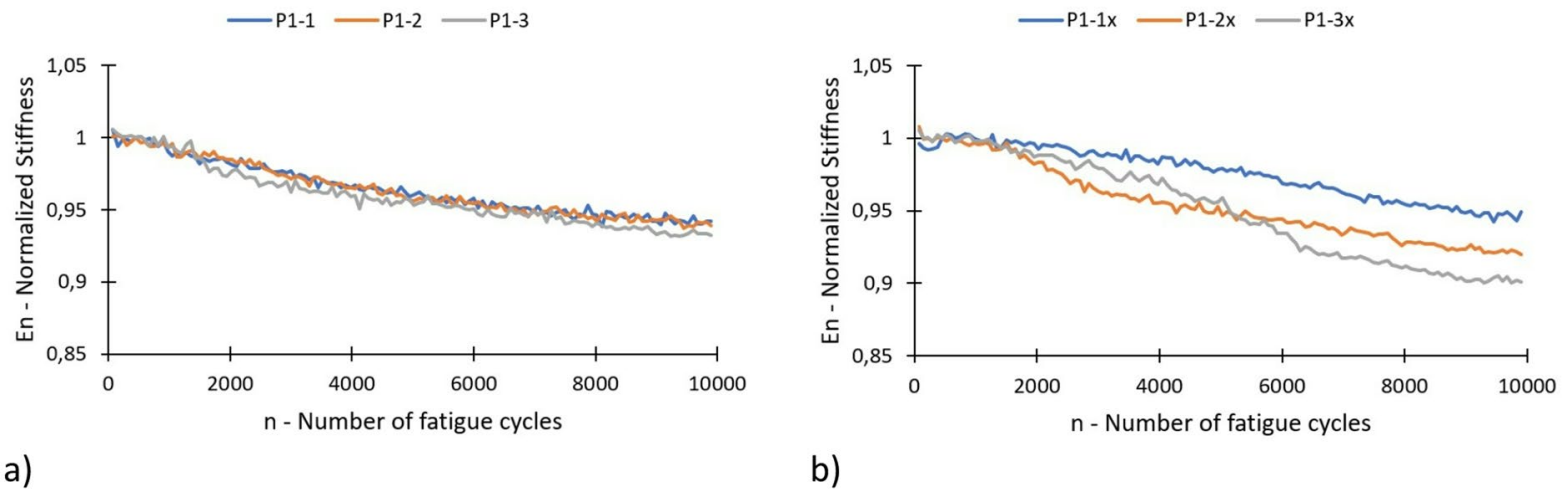
Degradation of specimens as a function of the number of cycles, represented by changes in normalised stiffness: (**a**) specimens with the V-shaped notch, (**b**) specimens with the U-shaped notch.

The initial value of normalised stiffness is 1, which is assumed to indicate an absence of damage to the specimen. This is obviously an approximation because the calculations start from a certain number of cycles rather than from the moment the test begins. As the material degrades, the normalised stiffness decreases. A value of E_n_ = 0 (which is practically unachievable) corresponds to complete failure of the specimen. Changes in the inclination of the normalised stiffness graph can be interpreted as changes in the rate of material degradation. If the angle of inclination from the horizontal is greater, then the degradation rate or level of damage is also greater. Graphs showing changes in normalised stiffness are presented in Fig. 6. Figure 6a shows the degradation of specimens marked as P1-1, P1-2, P1-3 (E_n_ ≈ 0.95), and Fig. 6b shows the degradation of specimens marked as P1-1x, P1-2x, P1-3 × (0.9 < E_n_ < 0.95).

Based on analyses of initial stiffness degradation, it was observed that specimens with the V-shaped notch showed higher convergence of results than specimens with the U-shaped notch. This more rapid initial stiffness decay in U-notched specimens often caused by the rapid formation of matrix micro-cracks, which tend to be more dispersed at the onset of loading due to the larger radius of the notch. Consequently, the U-notched specimens exhibited a more pronounced stiffness reduction at a given number of cycles during this phase.

In fibre‑reinforced composites, the fatigue response is influenced not only by notch geometry but also by other factors such as laminate manufacturing accuracy, layer orientation, and notch‑machining precision, which can contribute to the observed variability in stiffness reduction. A full assessment of the influence of notch type on fatigue life would require tests carried out to final failure, which were beyond the scope of the present study. Importantly, the V‑notched specimens exhibit damage initiation in a very specific and well‑defined region, and the subsequent propagation is more predictable compared to the U‑notched specimens. This results in higher convergence of the stiffness‑degradation curves for the V‑notch geometry, as also highlighted in the manuscript. In contrast, the semi‑circular notch promotes a broader stress redistribution zone, leading to more variable degradation patterns and larger scatter in stiffness decay. The objective of this work was to investigate the initiation and evolution of sub-critical fatigue damage and to evaluate the effectiveness of passive thermography and acoustic emission in monitoring such phenomena. Therefore, the observed differences between the notches should be interpreted strictly in the context of early-stage damage behavior rather than overall fatigue endurance.

**Fig. 7:**
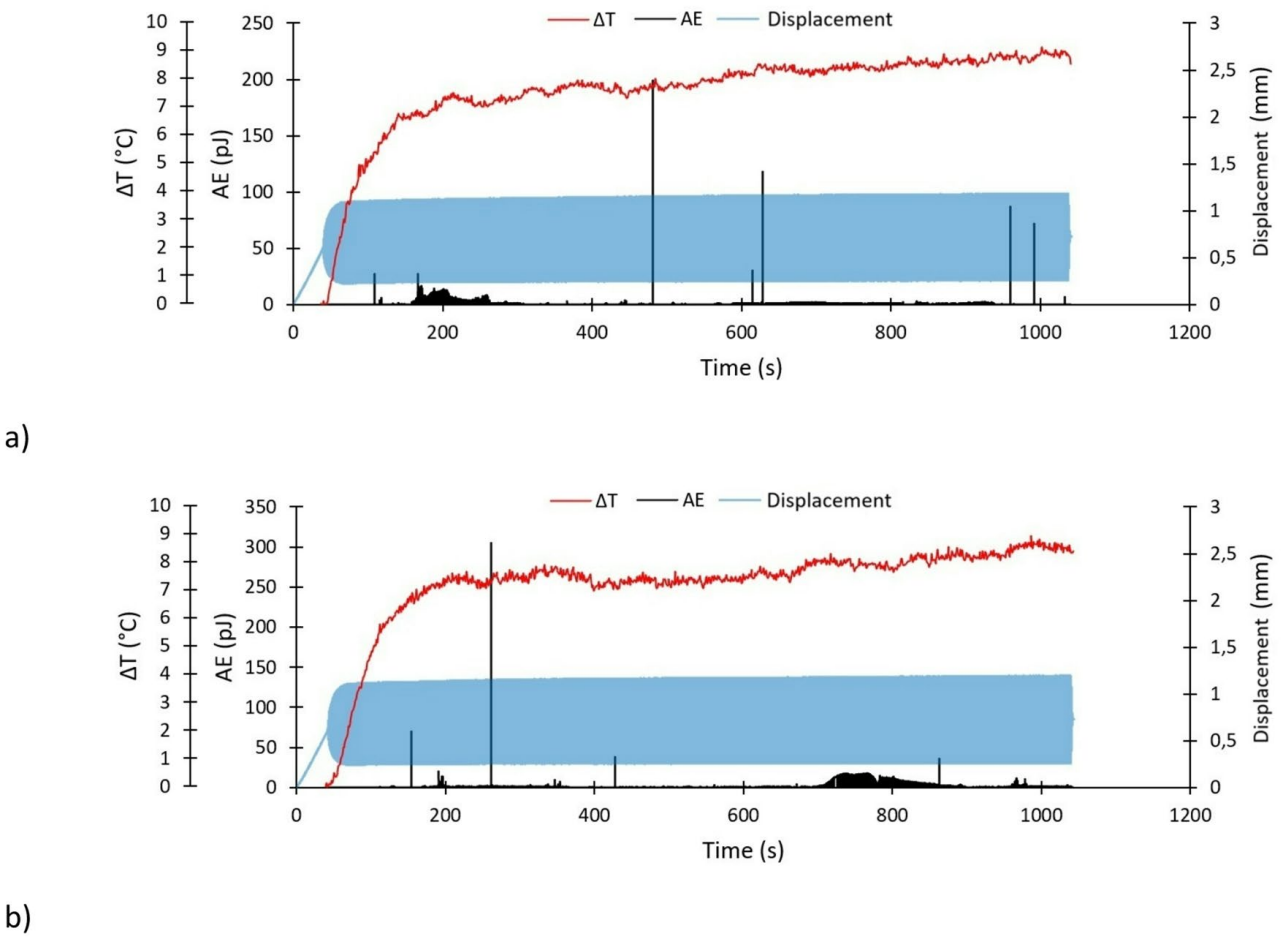
Experimental characteristics – specimens with the V-shaped notch: (**a**) P1-1, (**b**) P1-3.

**Fig. 8:**
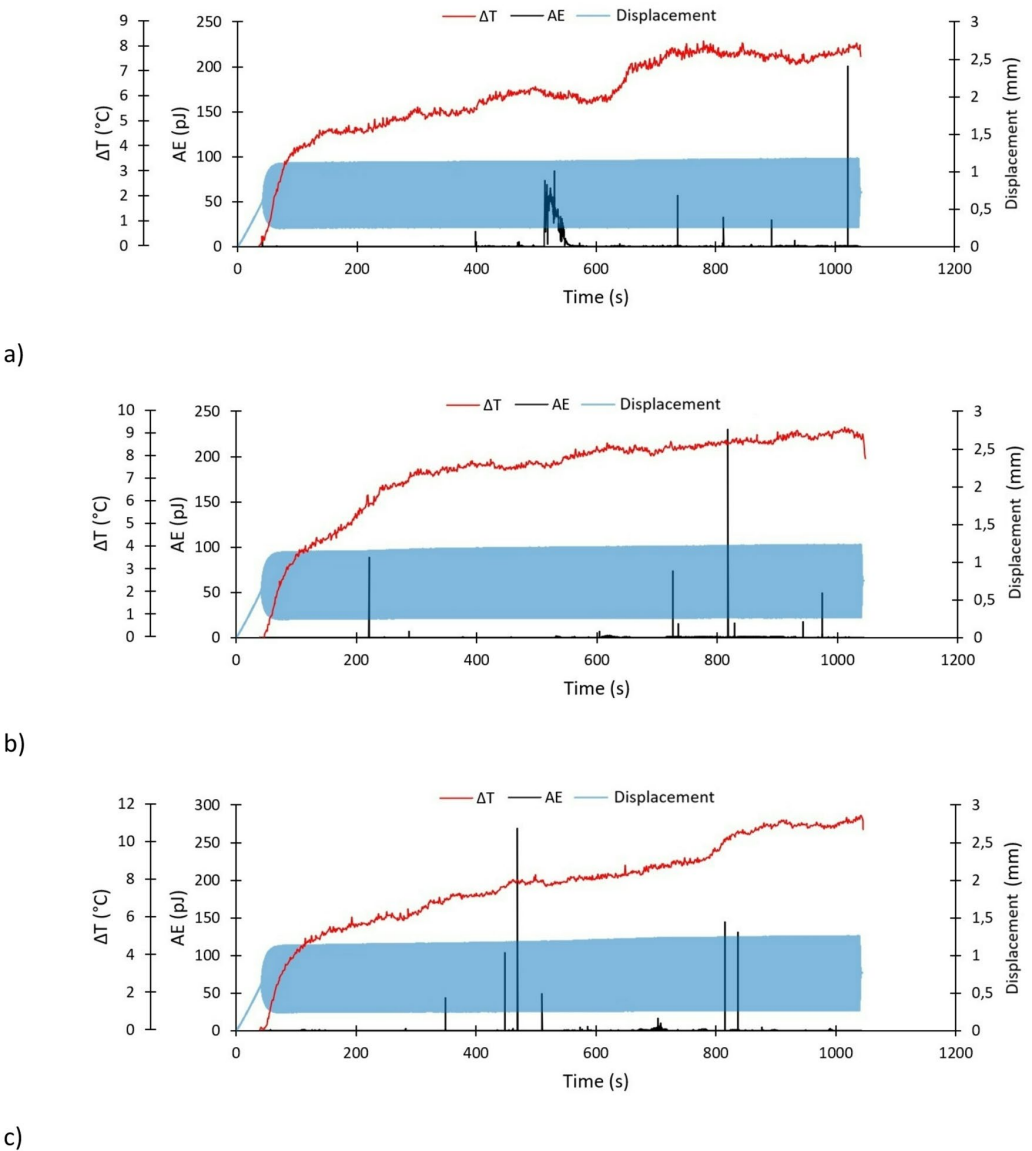
Experimental characteristics specimens with the U-shaped notch: (**a**) P1-1x, (**b**) P1-2x (**c**) P1-3x.

The two applied non-destructive testing methods enabled an independent assessment of damage during cyclic testing. In the case of passive thermography, the temperature increase (ΔT) curve could be determined in real time. Similarly, acoustic emission made it possible to record selected acoustic emission signals (primarily the energy signal) in real time. The results obtained from both non-destructive testing methods were then compared with the deformation characteristics of the fatigue-tested specimens (elongation measured in real time). This methodological approach enabled a comprehensive quantitative assessment of the strength of the tested structures. Based on this approach, taking into account the real-time evolution of specimen elongation, temperature increase (ΔT) and acoustic emission energy signal (Figs. 7 and 8). During testing, no valid acoustic emission and thermography signals were recorded for specimen P1-2; therefore, the corresponding results were omitted.

The Figs. 7 and 8 show the complete test data, which consist of the specimen tensioning (to an average fatigue load value F_m_) and the target fatigue test. Due to the fact that the first damages appeared only after the fatigue tests were started, thermography and acoustic emission measurements were started at the same time as the fatigue tests had started. Both signals were recorded independently.

The parameters presented in the legends of Figs. 7 and 8 are as follows: ΔT – temperature increase, AE – acoustic emission energy signal, Displacement – specimen elongation. For specimens P1-1 and P1-3, a temperature increase of approximately 7 °C was observed in the initial phase of fatigue testing (from the beginning of the test to approximately 200s). From 200 s to the end of the test, a further slight increase in temperature was observed, ranging from 7 to 9 °C. In the case of acoustic emission energy signal analysis, local increases in the energy signal were observed, indicating the initiation of damage to the composite material. For specimen P1-1, the maximum signal increase was observed at 481s (199 pJ). Other significant increases in the signal occurred at 628s (118 pJ), 959s (87 pJ) and 992s (72 pJ). During the remainder stage of the tests, increases in the acoustic emission signal were negligible. For specimen P1-3, the maximum signal increase was observed at 262s (305 pJ). Other significant increase in the signal occurred at 155s (70 pJ). For both specimens, the acoustic energy signal become dense within 200th second of the test. Despite the signal being weak, that event is not negligible and it might be an indication of the first damage to the specimen. During the remainder stage of the test (apart from those indicated above), the increases in acoustic signal were negligible.

The situation was slightly different in the case of signals recorded for specimens P1-1x, P1-2x, and P1-3x. It was observed that the temperature increase was more intense throughout the duration of the tests. In the initial range, the temperature increase was lower than in the case of specimens with the V-shaped notch – at 200s, a temperature increase of approximately 5–6 °C was observed. The maximum temperature rise during the tests was contained in the range of 8–11.5 °C. In the case of acoustic emission energy signal analysis, local peaks in the energy signal were observed, indicating the initiation of damage to the composite material. For specimen P1-1x, the maximum signal increase was observed at 1021s (201 pJ). Other significant increases in the signal occurred at 530s (84 pJ) and 737s (57 pJ). For specimen P1-2x, the highest peak was observed at 818s (230 pJ) followed by increases at 221s (88 pJ) and 726s (73 pJ). For specimen P1-3x, the maximum occurred at 469s (269 pJ), with additional peaks at 448s (104 pJ), 815s (144 pJ) and 837s (131 pJ). During the remainder stage of the test, the increases in acoustic signal were negligible—the issue concerns all specimens.

In the case of acoustic energy emission signal analysis, only signals exceeding 50 pJ were taken into account. Individual signals with lower values did not demonstrate any significant changes in the behaviour of the tested composite structures. No significant similarities were observed in the acoustic emission energy signal course of any of the specimens. However, local ‘peaks’ in the signal were observed in each specimen, indicating the possibility of damage initiation to the composite structure being subjected to fatigue testing. Based on the test results, significant differences in temperature increase were observed in specimens with the V and U-shaped notch. In specimens with the V-shaped notch, the temperature increase stabilised at 200s, after sharp increase in the first stage of the test, showing a nearly linear trend afterwards. In contrast, specimens with the U-shaped notch demonstrated a significant non-linearity in temperature increase throughout the entire range. In the case of registered elongation of specimens using an MTS fatigue machine, almost identical behaviour was observed, regardless of the type of notch. During the tensioning process, which occurred from the beginning of the tests to approximately 40s, the specimens were elongated by approximately 0.6 mm. During fatigue testing, the maximum recorded elongation values were as follows: P1-1—1.19 mm, P1-3—1.20 mm, P1-1x—1.18 mm, P1-2x—1.23 mm, P1-3x—1.26 mm.

**Fig. 9:**
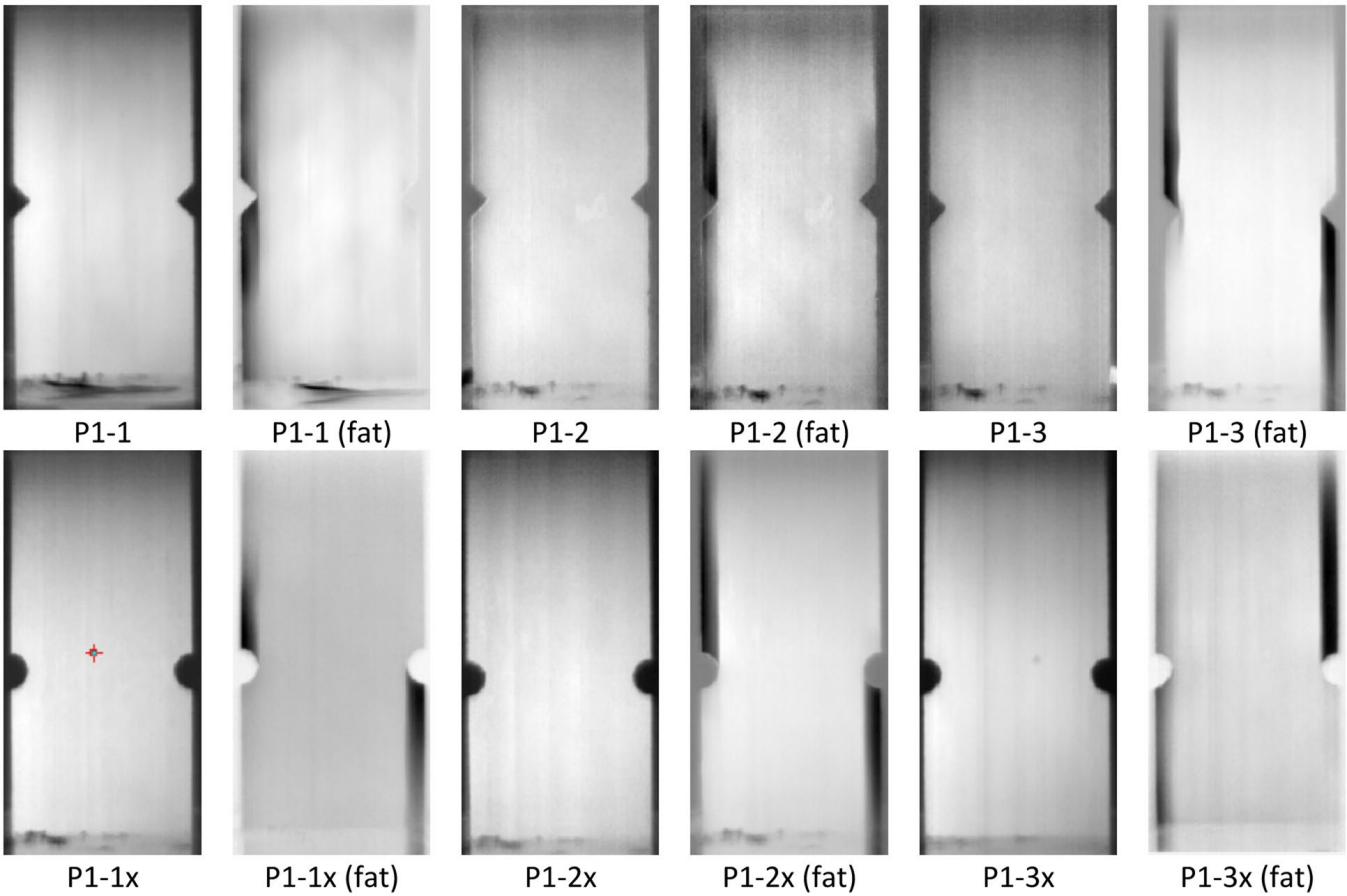
Thermal images of analysed specimens before and after fatigue tests (fat).

Figure 9 presents the thermal images of analysed specimens before and after fatigue tests (fat). After fatigue tests, the longitudinal separations of outside layers (0) is visible in almost all analysed specimens. The observed delamination affected the outer layers and most frequently occurred asymmetrically in areas above or below the notches on both sides of the specimen, with a width equal to that of the notch.

**Fig. 10:**
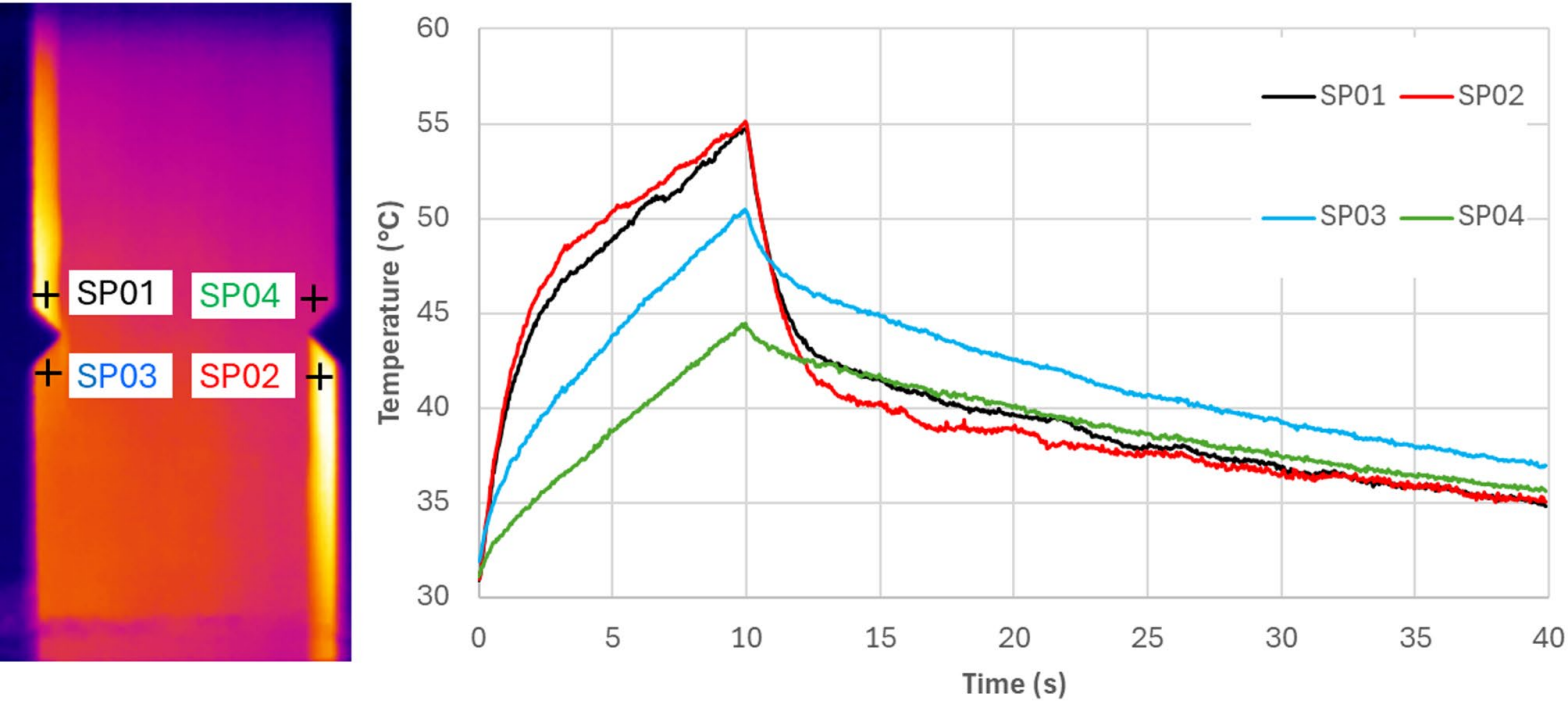
Representative thermal analysis after fatigue test in an active mode for the specimen P1-3.

Figure 10 shows the representative active thermographic analysis of the P1-3 specimen after the fatigue test. Points SP01 and SP02 indicate the areas of damage after the fatigue testing. A thermal image presents the specimen during the heating in 3s of the analysis. The visible difference in temperature of the specimen indicates the existence of defects after fatigue loading. During sample heating, the greatest temperature differences between damaged and undamaged areas are visible in active thermography.

**Fig. 11:**
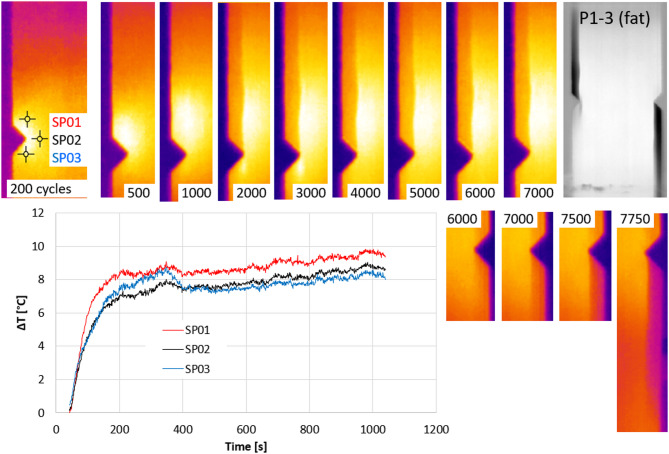
The local thermographic effects during the fatigue tests of the specimen P1-3.

**Fig. 12:**
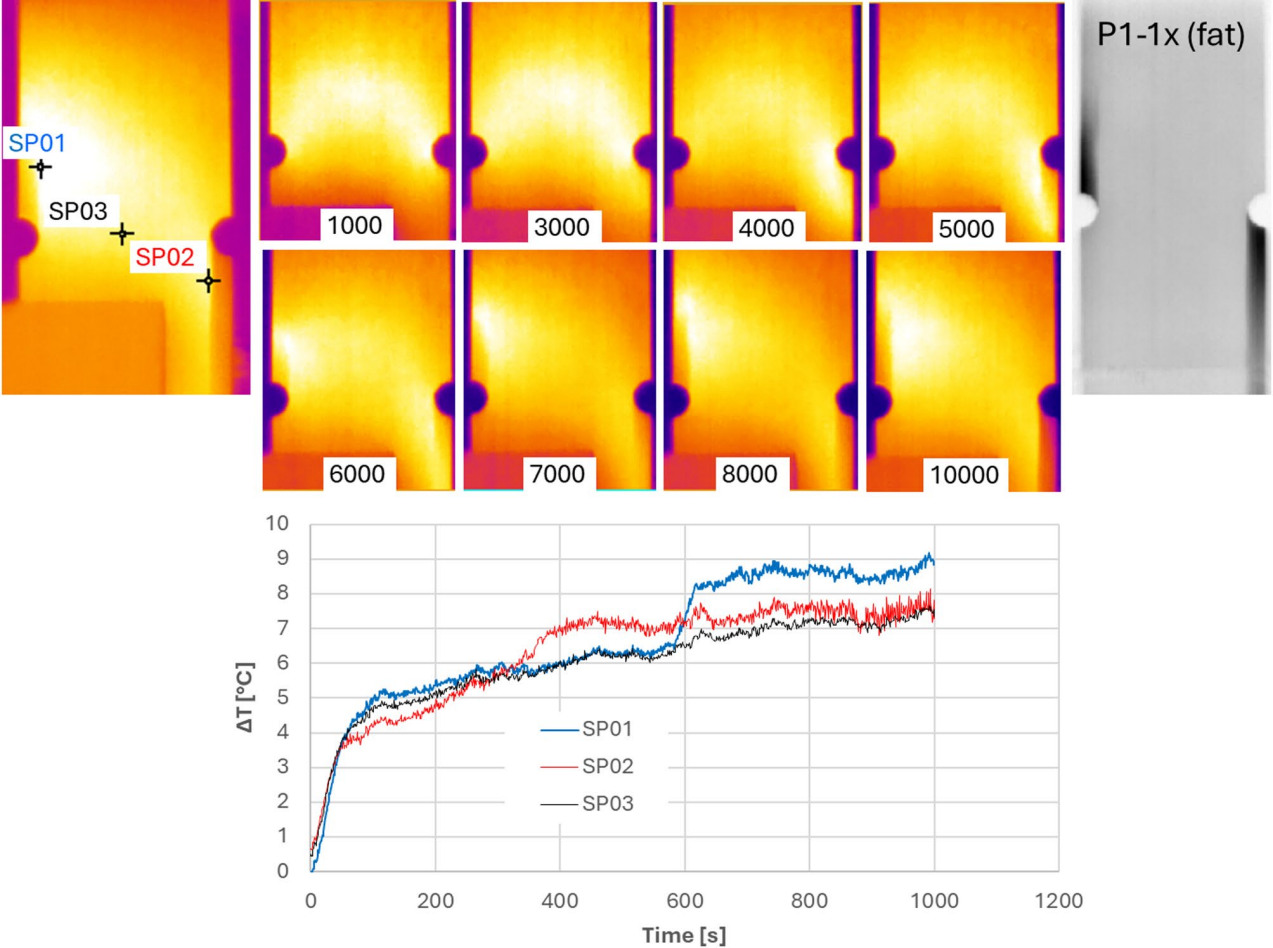
The local thermographic effects during the fatigue tests of the specimen P1-1x.

The passive thermography was conducted during the fatigue tests. The selected thermographic results of the specimen P1-3 with the V-shaped notch and the specimen P1-1x with the U-shaped notch are presented in Figs. 11 and 12. Considering the fatigue‑test frequency of 10 Hz, the number of cycles can be easily estimated by dividing the cycle count by 10 to obtain time in seconds.

A crack appears above the left notch between 1200 and 1300 cycles. By 2000 cycles, the crack has reached the top of the specimen. After approximately 4000 cycles, the effects of fatigue loading become visible in the area around the right notch. Clearly, the damage propagates from above the left notch (SP01) from the outset. Between 2000 and 3000 cycles, localised damage appears below the left notch (SP03), but this does not propagate further. At around 6200 cycles, considerable damage occurs below the right notch. Between 7400 and 7500 cycles, a crack appears below the right notch and quickly (within 300 cycles) reaches the lower part of the sample.

The local temperature difference between adjacent points at the three locations indicated on the specimen P1-3 shows the timing and dynamics of the fatigue process. The sudden fracture released a large amount of energy in the form of a temperature increase, which occurred almost simultaneously near and above the left notch. From this point onwards, little happens on the left side of the specimen. Progressive degradation can then be seen below the right notch, leading to sudden damage. As the load-carrying capacity of the material below the right notch is reached, a sudden fracture occurs below the notch. This is followed by an equilibration of temperatures. Once significant delamination has formed above the left notch and below the right notch, the damaged areas can no longer effectively transfer load. At this stage, the remaining intact part of the specimen is the central section between the notches, which temporarily carries the majority of the load.

As can be seen in Fig. 9, the final form of the damage of specimen P1-1x is the same as in the case of the first notch, but the manner of the material’s degradation is different. In the first stage of degradation, comparing SP01 with SP02 (but also observing the gradual increase in temperature at point SP03), it is visible how the ± 45° layers work, although the temperature increase began locally at the notches, the sample works throughout the entire central part in accordance with the ± 45° layer arrangement and not, as in the case of the previous notch, only vertically. At 2500–3500 cycles, the temperatures at the analysed points equalise and stabilise. This is because the degradation level of the ± 45° layers has been reached and the load has been transferred to the 0° layers. Only in the later stages, as degradation of these layers progresses, do the 0° layers begin to play a greater role, with damage appearing at the notches. Degradation occurs in the area under the right notch (SP02). The damage that appears after approximately 3500 cycles gradually propagates up to 5000 cycles. Similarly, gradual degradation occurs in the area above the left notch (SP01), with visible damage appearing after around 6000 cycles and spreading quickly upwards.

Unlike the global effects presented in Figs. 7 and 8, the temperature characteristics shown in Figs. 11 and 12 enable a localized description of the increasing degradation of the analyzed specimens resulting from fatigue loading. Comparing the temperature curves of sample P1-3 in Figs. 7 and 11 reveals that the global temperature change (Fig. 7) only considers temperature changes at point SP01 (Fig. 11), where the highest temperature were recorded. The local temperature characteristics shown in Fig. 11 allow for more precise monitoring of damage progression in the specimen. A local approach reveals changes related to damage development in different areas of the sample, as well as a reduction in the load-carrying capacity of individual layers. Comparing the global characteristics (Fig. 8) with the local characteristics (Fig. 12) for the specimen featuring the U-shaped notch reveals equally interesting effects. As the damage in specimen P1-1x developed more dynamically (Fig. 12), the global approach could not capture this effect. Initially, the global characteristics of specimen P1-1x (Fig. 8) show damage forming above the left notch, followed by dynamic damage formation below the right notch after 3500 cycles. After 6000 cycles, there is a rapid increase in damage above the left notch.

Based on the tests conducted, certain differences in the behaviour of specimens featuring two different types of notch were observed. In terms of stiffness changes (Fig. 6), specimens with a V-notch were shown to exhibit a smaller average decrease in stiffness (approximately 5%) than those with a U-notch. Using the acoustic emission method, it was observed that specimens with a V-notch showed slightly smaller local peaks in the energy signal, indicating damage initiation in the composite material. Local increases in the acoustic energy signal occur in specimens with a V-notch as early as the initial phase of fatigue testing (before 200 s), whereas in U-notched specimens, such increases typically emerged in a later stage. Furthermore, a higher acoustic energy signal intensity (especially in local peaks) is observed in specimens with U-shaped notches. The stiffness degradation parameter (Fig. 6) reflects the global reduction in stiffness at a given number of cycles, which is consistent with the global temperature rise recorded via passive thermography (Figs. 7 and 8). However, local thermographic analyses (Figs. 11, 12) reveal that the notch geometry significantly influences local failure mechanisms; specifically, more intense localized degradation was observed in the vicinity of the V-notch, even though the overall global stiffness decay remained lower for this geometry.

**Fig. 13:**
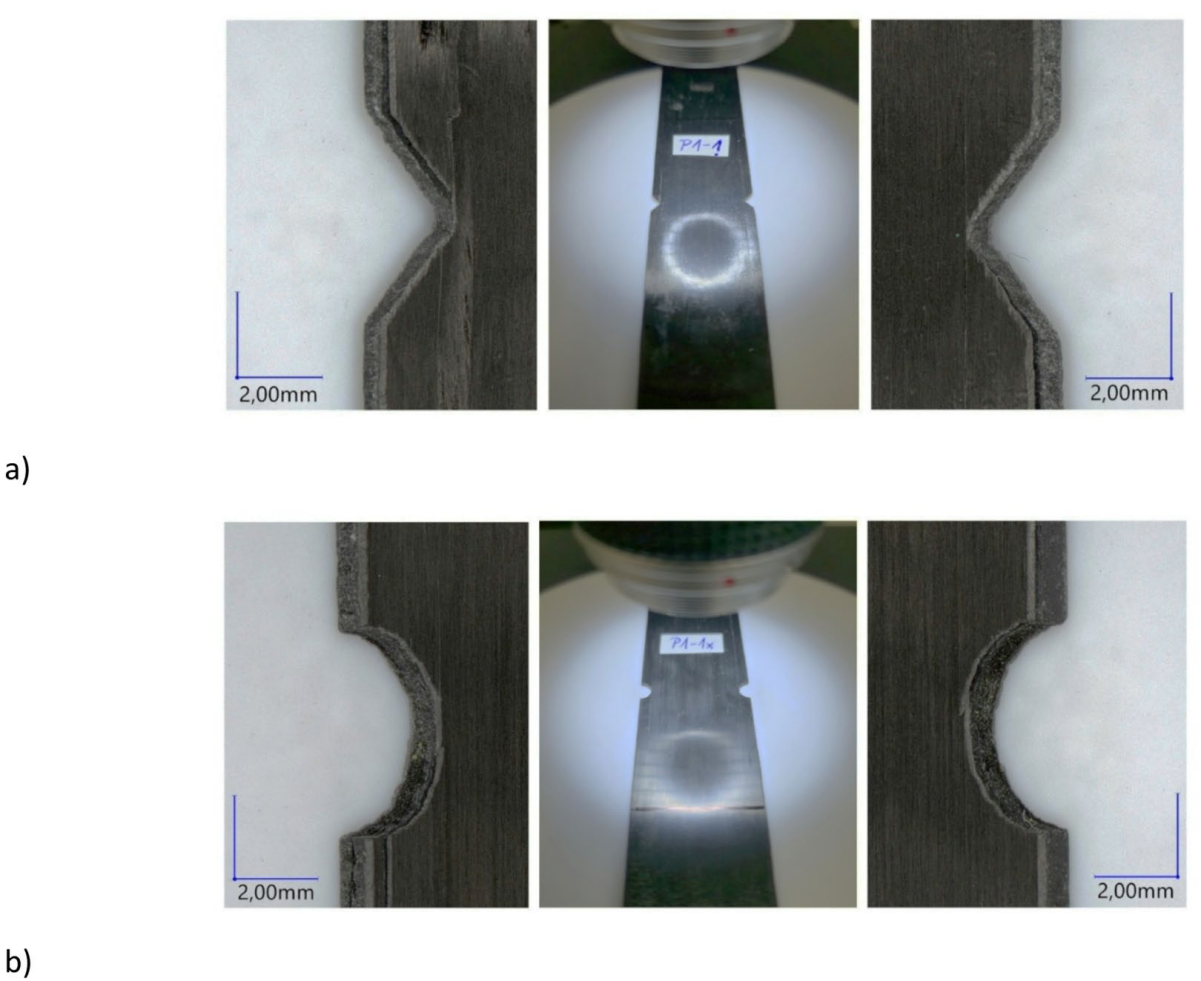
Microscopic evaluation of damage: (**a**) specimen P1-1, (**b**) specimen P1-1x.

The final stage of the analysis involved research based on digital microscopy. During this research, a qualitative assessment of the damage to composite structures subjected to fatigue testing was conducted using a digital microscope. As part of the research, more detailed observations of the damaged areas were carried out. The use of depth of field options enabled a more accurate representation of the damage—Fig. 13.

**Fig. 14:**
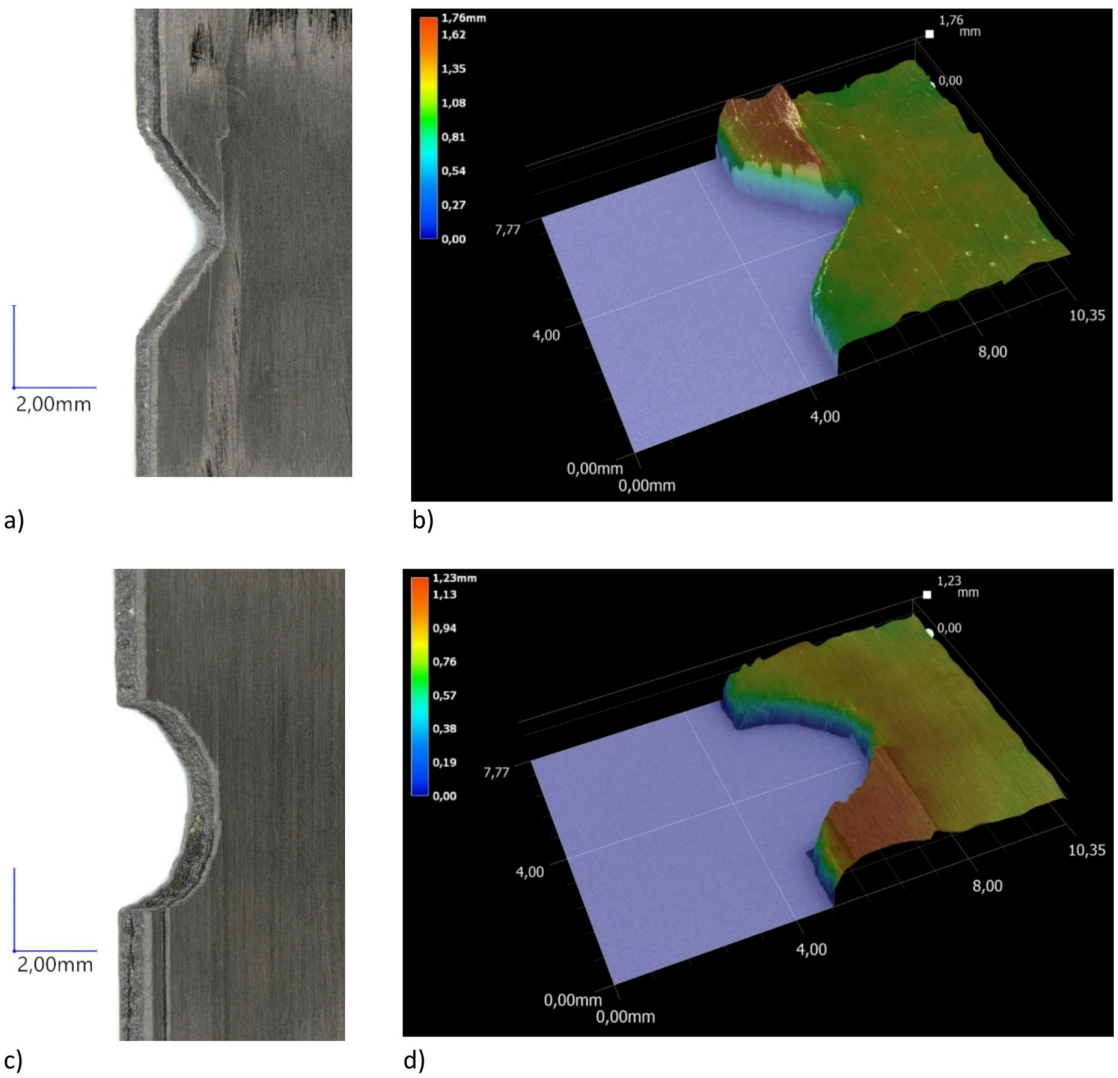
Detailed microscopic observation of the damage: (**a**) 45° tilt angle of microscope head (P1-1), (**b**) 3D visualization of the damage area (P1-1), (**c**) 45° tilt angle of microscope head (P1-1x), (**d**) 3D visualization of the damage area (P1-1x).

Figure 14 shows the different types of damage in more detail, providing a more accurate reflection of the nature of progressive damage.

Microscopic examinations were conducted to observe damage to composite structures subjected to fatigue testing. The research identified that the predominant forms of damage occurred primarily in the notch area. In all tested specimens, the damage consisted of delamination of the outer plies of the composite throughout the width of the notches, demonstrating progressive damage along the longitudinal direction of the specimens. During fatigue testing, damage occurred in the form of vertical delamination beginning at the notch and propagating along the specimen’s length (longitudinal direction)—Fig. 13. The tilt angle of the recording head in relation to the specimens being observed was set at 45°. Microscopic analysis was performed at 20x magnification. Depth composition was used to register all captured forms of damage, making it possible to capture areas of damage with precise focus adjustment. Digital microscopy revealed a local rupture in the connection between the outer plies of the composite at the notches. In all cases, regardless of notch type, vertical cracks were observed in the outer layers of the composite. These cracks propagated from the end of the notch. The predominant forms of damage affected the outermost plies of the composite structures, with no significant damage observed in the transverse direction. Digital microscopy observations show that the type of notch had no significant effect on the nature of the damage observed in the composite structures. In the context of a qualitative assessment of damage using digital microscopy, more detailed observations were made of selected specimens. For this purpose, defects were observed in greater detail using higher magnification (30x) of the damaged areas, and 3D visualizations of the damaged areas were generated—Fig. 14. The 3D visualisation considered the thickness of the specimens (0.84 mm), and the areas marked in red (the maximum value on the legend) represented delamination of the outer layers of the composite. The 3D visualisations were intended to show the damaged areas of the test specimens. In summary, the forms of damage were similar in all analysed cases, regardless of the type of notch. The damage phenomenon demonstrated a scientifically significant common feature in all of the test specimens. Each specimen was damaged in a relatively similar manner, with the damage occurring diagonally, on the opposite sides of the notches. While the damage propagated upwards from the left notch of the specimen, it propagated downwards from the right notch. Delamination occurred in the notch zone of each of the analysed cases, taking the form of vertical strips that constituted fragments of the laminate’s outer layers.

## Conclusions

The research confirms that combining infrared thermography and acoustic emission provides a comprehensive diagnostic perspective on the initiation and development of early-stage fatigue damage in thin-walled CFRP specimens with different notch geometries. A qualitative assessment and validation of NDT observations of the damage in composite structures subjected to fatigue testing was conducted using a digital microscope. The study demonstrates that both global and local thermal effects, together with AE-based damage indicators, form a coherent set of complementary diagnostic features that reliably describe damage behaviour in composite laminates. The conclusions obtained from the study can be divided into groups related to: (i) the influence of notch geometry, (ii) thermal responses (global and local), (iii) acoustic‑emission‑based indicators, and (iv) microscopic validation.

Influence of notch geometry.

- Specimens with U‑shaped notches exhibit a greater decrease in normalised stiffness within 10,000 cycles than specimens with V‑shaped notches, indicating stronger structural degradation in the early phase of fatigue loading.
- Despite differences in stiffness evolution and thermal response, the qualitative form of damage remains similar for both geometries: vertical delamination of the outer plies initiated at the notch and propagating along the specimen length.
- V‑notched specimens show more predictable localisation of damage initiation, whereas U‑notched specimens exhibit more spatially distributed early degradation due to their lower stress concentration radius.

Global thermal effects.

- Global passive thermography reveals that V‑notched specimens stabilise their temperature rise earlier, followed by a nearly linear trend, while U‑notched specimens display continuous non‑linear ΔT growth.
- Maximum global temperature increases differ based on notch geometry: approx. 9 °C for V‑notches and approx. 11.5 °C for U‑notches. These global temperature‑response differences correlate with global stiffness degradation trends, confirming that thermography effectively reflects structural‑scale material weakening.

Local thermal effects.

- Local thermographic monitoring shows that fatigue damage does not develop uniformly.
- In V‑notched specimens, temperature rises are concentrated above the notch at early stages, indicating a sharply localised damage front.
- In U‑notched specimens, thermal activity develops more gradually and across a wider region, confirming a more distributed stress‑redistribution zone characteristic for larger notch radii.
- Local temperature monitoring therefore provides a more sensitive description of the early‑stage degradation.

Acoustic emission (AE) response.

- AE measurements consistently detect energy peaks associated with damage initiation.
- V-notched specimens generate early, lower-intensity energy peaks, reflecting strongly localised damage initiation.
- U-notched specimens produce later but more intense AE peaks, consistent with globally observed higher ΔT and greater stiffness loss within 10,000 cycles.

Digital microscopy—validation of NDT observations.

- Microscopic analysis validates both thermographic and AE indicators and confirms that the primary form of damage is delamination of the outer layers initiated at the notch root.
- 3D visualisation techniques reveal the extent of delamination and confirm that diagonal propagation paths on opposite sides of the notches are consistent across all specimens.
- The microscopy results reinforce the conclusion that notch geometry influences the rate and spatial distribution of damage, but not its qualitative form.

Overall, the findings of this study show that the combined infrared thermography, acoustic emission, and digital microscopy provides a coherent, multi-scale description of early-stage fatigue damage in notched CFRP laminates. This integrated diagnostic approach enables early detection, localisation, and qualitative identification of failure mechanisms that would not be observable using a single technique alone. Based on these outcomes, future work will extend the experimental programme towards tests carried out to final failure, investigate the influence of laminate stacking sequence on multi‑scale diagnostic responses, and develop numerical models coupling FEM with thermographic and acoustic‑emission data. These efforts aim to further strengthen the predictive capability and practical applicability of non‑destructive diagnostics in the fatigue strength assessment of composite structures.

## Data Availability

The data that support the findings of this study are available from the corresponding author, upon reasonable request.
